# A computational study of visual working memory capacity in the presence of saliency effects

**DOI:** 10.1186/1471-2202-12-S1-P64

**Published:** 2011-07-18

**Authors:** Laura Dempere-Marco, David P Melcher, Gustavo Deco

**Affiliations:** 1 Department of Information and Communication Technologies, Universitat Pompeu Fabra, 08018 Barcelona, Spain; 2 Department of Cognitive Sciences and Education, University of Trento, 38068 Rovereto, Italy; 3 Institució Catalana de Recerca i Estudis Avançats, Universitat Pompeu Fabra, 08010 Barcelona, Spain

## 

The study of working memory capacity is of outmost importance in cognitive psychology as working memory is at the basis of general cognitive function. Although the storage capacity limit has been thoroughly studied [1], its origin still remains a matter of debate. Several neurophysiological studies suggest that items are maintained in working memory through elevated firing activity in cortical neural assemblies that selectively respond to specific stimuli. Accounting for this experimental observation, Edin et al. [2] proposed a mechanistic explanation of the top-down control of working memory capacity and, based on a mean-field analysis, established an upper boundary to the number of items that can be held in memory. By also making use of a recurrent network model of working memory [3], we have further investigated the mechanisms underlying working memory capacity while also accounting for the two following experimental observations: 1) visual saliency reduces the number of items that can be held in working memory, and 2) visually salient items are commonly kept in memory in delay match-to-sample tasks at the cost of not keeping as many non-salient items.

**Figure 1:**
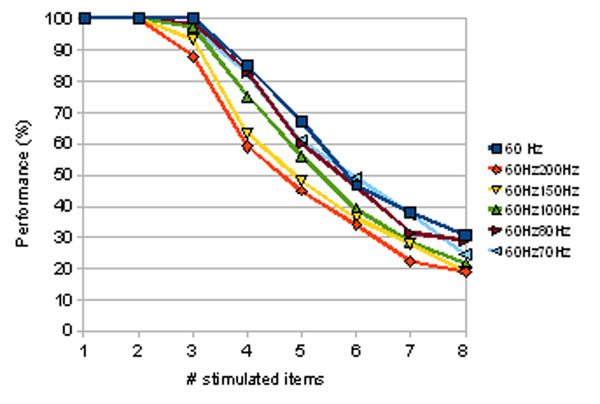
Theoretical prediction of the performance derived from computational simulations of a delayed match-to-sample task with *n* selective neural assemblies simultaneously stimulated. Performance is calculated by assuming that an item is held in memory when its associated selective pool shows a mean persistent activity ν_thres_ ≥ 20 Hz during a period of 2 s after removal of the stimuli. *n*-1 selective pools are stimulated at λ = 60 Hz and the remaining pool receives a higher stimulation λ_sal_ that emulates visual saliency. λ_sal_ = 60 Hz (no saliency), 70 Hz, 80 Hz, 100 Hz, 150 Hz and 200 Hz. The results are derived from computational simulations of a biologically plausible network of spiking neurons [3].

## Conclusions

A severe limitation in visual working memory capacity arises from the constraints that lateral inhibition imposes to the mnemonic activity during the delay period. However, it is also worth noting that a further limitation to working memory capacity derives from the need that the neural assemblies which have received stimulation reach a sufficiently high level of excitation. In particular, in the presence of visual saliency, the neural assemblies that receive stimulation but are not selective to the salient stimulus are less likely to achieve the elevated firing rates required to be subsequently held in memory.

## References

[B1] Cowan N The magical number 4 in short-term memory: A reconsideration of mental storage capacity Behav Brain Sci 2000 24 87 114 10.1017/S0140525X01003922 11515286

[B2] Edin F Klingberg T Johansson P McNab F Tegnér J Compte A Mechanism for top-down control of working memory capacity Proc Natl Acad Sci 2009 106 16 6802 6807 19339493 10.1073/pnas.0901894106 PMC2672558

[B3] Brunel N Wang XJ Effects of neuromodulation in a cortical network of object working memory dominated by recurrent inhibition J Comput Neurosci 2001 11 63 85 10.1023/A:1011204814320 11524578

